# Pegylated Interferon Alpha for Chronic Hepatitis B Virus Infection

**DOI:** 10.1155/2022/3890309

**Published:** 2022-01-06

**Authors:** Joana Vasconcelos, João Domingos, Lia Bastos, Teresa Baptista, Kamal Mansinho

**Affiliations:** Infectious Diseases Department, Hospital Egas Moniz, Centro Hospitalar Lisboa Ocidental, Lisboa, Portugal

## Abstract

Chronic hepatitis B (CHB) is a potentially life-threatening and prevalent disease worldwide. Far from attaining the ultimate treatment goal, hepatitis B virus (HBV) infection eradication, the two current therapeutic options aim to prevent progression to end-stage liver disease, maintaining long-term suppression of HBV replication. Pegylated interferon-*α* (PEG-INF*α*) is often poorly tolerated and disregarded considering the orally administered nucleos(t)ide analogues. However, PEG-INF*α* may achieve similar treatment endpoints with a finite course of treatment. We report a case of PEG-INF*α*-treated CHB that attained sustained off-treatment virological response with only 16 weeks of treatment, with loss of both HBeAg and HBsAg (this latter the optimal treatment endpoint).

## 1. Introduction

Chronic hepatitis B (CHB) remains a highly prevalent infectious disease worldwide, affecting circa 240 million people [1]. Hepatitis B virus (HBV) is the main leading cause of CHB, cirrhosis, and hepatocellular carcinoma, causing 887000 deaths in 2015 [2].

Two first-line therapeutic options have been contemplated in Asian-Pacific, American, and European guidelines [1, 3, 4]: nucleos(t)ide analogues (NAs) and pegylated interferon-*α* (PEG-IFN*α*). Far from attaining the ultimate treatment goal (HBV infection eradication), the current and more realistic objectives involve improving quality of life and survival of HBV-infected people, preventing the progression to end-stage liver disease [1, 3, 4]. In order to do so, HBV replication should be sustained and long-term suppressed below the threshold of liver injury.

The optimal CHB treatment endpoint, although rarely achieved, is sustained off-therapy loss of HBsAg, with or without seroconversion, representing a “functional cure.”. This associates with remission of HBV activity, improving long-term prognosis [1]. A commoner but less reliable treatment endpoint is loss of HBeAg in HBeAg-positive CHB, but a more realistic, and thus, the cornerstone endpoint of current CHB treatment is viral suppression [3, 4]. PEG-INF*α*, which has both immunomodulatory and antiviral properties, has the potential advantage to be able to achieve it with a finite course of treatment (usually 48 weeks). It has been used for the treatment of CHB for more than 25 years, and its pegylated form made weekly administrations possible [5]. There is no drug resistance, and a higher rate of HBeAg and HBsAg seroconversion has been reported [6–9]. However, it is often poorly tolerated since it is an injectable treatment with multiple adverse side effects: fever, myalgias, headache, asthenia, cytopenia, hair loss, mood alterations (including depression), and the development of autoantibodies, namely, antithyroid [5]. On the contrary, recent NAs have high barrier to resistance, they are orally administered, very-well tolerated, and ultimately serologic responses may approach the ones of PEG-INF*α* throughout the years, eventhough indefinite treatment should be anticipated unless certain treatment endpoints are met [5, 8, 9].

We report a case of PEG-INF*α*-treated CHB, with loss of both HBeAg and HBsAg, with only 16 weeks of treatment.

## 2. Case Presentation

A 47-year-old Brazilian man living in Portugal came to our infectious diseases (ID) outpatient clinic, referred from his general practitioner after being diagnosed with hepatitis B (routine testing). Previous medical conditions included hepatitis A (in his childhood). He was not under any medication. There was no previous smoking, alcohol, or intravenous drugs consumption history. No allergies were reported. He denied any previous surgeries; he had no tattoos. He was homosexual, being married for 12 years, although having a nonmonogamous relationship. Although always asymptomatic, he had been previously tested for HBV 10 years before coming to the ID clinic, since his husband had an acute hepatitis B (AHB) episode. By then, he had no serologic markers of previous infection or immunization (he also denied HBV vaccination). He was proposed HBV vaccination but ended up not doing it. He repeated an HBV test 20 months before, while in Brazil, he showed us that it revealed positive HBsAg, HBeAg, HBsAb, and HBcAb, negative HBeAb. He did not look for medical assistance during that time and he denied recalling any AHB symptoms. At presentation, his laboratory results showed HBV DNA 404 × 10^6^ IU/ml and positive HBsAg (index 598), HBsAb, HBeAg, and total HBcAb were with a negative IgM HBcAb. Hepatic tests revealed elevated ALT (118 IU/L, >2x upper limit of normal (ULN)) and AST (62 IU/L). GGT, ALP, INR, bilirubin, and albumin were within normal values. HIV, HCV, and treponema-specific test were negative. No abnormalities were noted in hemogram, leukogram, platelets, renal and thyroid tests, and ionogram. Dyslipidemia and hyperglycemia were analytically excluded. Abdominal ultrasonography was unremarkable. CHB was assumed (persistent HBsAg for 20 months), and he was proposed PEG-IFN*α* 180 *µ*g weekly injections. He completely adhered and tolerated treatment for the first weeks, but he started to feel increasing asthenia during the course of treatment, with no other meaningful symptoms. His 12 weeks' analyses revealed neutropenia (720 cells/*µ*L) and thrombocytopenia (81.000 platelets/*µ*L), AST 227 IU/L, ALT 404 IU/L, subclinical hypothyroidism (TSH 7,79 *µ*UI/mL; T4 13,6 pmol/L), and positive HBsAg (index 33,2), HBsAb, HBcAb, and HBeAb, with loss of HBeAg, HBV DNA 237 (log_10_2.37). Despite his remarkable response, due to bicytopenia and his reported intolerability to treatment, treatment suspension for 2 weeks was agreed. After complete analytical and clinical improvement, PEG-IFN*α* was reintroduced at a lower dose (90 *µ*g weekly), and the patient was reevaluated 4 weeks after. By then, he was again reporting asthenia and was unwilling to maintain treatment. Analytically, DNA HBV < 20 IU/L, loss of HBsAg, negative HBeAg, and positive HBeAb and HBsAb. Considering intolerable side effects and accomplishment of the optimal therapy endpoint (loss of HBsAg), treatment was stopped (a total of 16 weeks was completed). Six months after discontinuation therapy, he was asymptomatic and remained virologically suppressed, maintaining negative HBsAg and a completely normal hepatic panel (AST 38 IU/L, ALT 32 IU/L). Twelve months after discontinuation therapy, sustained off-treatment virological response was confirmed (negative HBsAg, viral load HBV < 10 IU/mL, AST 29 U/L, and ALT 29 U/L). The abovementioned evolution is given in Table 1.

## 3. Discussion

We report a case of CHB with a dramatic response to PEG-IFN*α*. This is a representative case of both strengths and weaknesses of this 1^st^ line treatment.

We were first presented with a young man with no previous comorbidities and a diagnosis of HBeAg-positive CHB. At admission, he had a striking elevated viral load (VL) and high aminotransferases (ALT > 2x ULS), which configured therapeutic indication. He was most willing to adhere to a finite course of treatment, and he did not mind the weekly injectable administration of PEG-IFN*α*, which he did while at home.

Some baseline factors have been reported to predict sustained off-treatment response with PEG-IFN*α* in HBeAg-positive CHB [3, 7]; curiously, our patient only had one of them (high ALT). We did not determine HBV genotype (A and B are associated with a better response), but low VL, older age, and female gender (also predictors of good response) were not verified.

We were only able to accomplish a course of 16 weeks of treatment, which is significantly lower than what has been suggested in guidelines (48 weeks) [1, 3, 4, 9]. However, there are some reports of comparable HBeAg seroconversion in similar populations with both 24 and 48 weeks of treatment [10–12], and further investigation is needed to determine if in particular cases (considering predictors of response) it is possible to shorten treatment's duration. One of the main pitfalls of PEG-IFN*α* is its low tolerability; in this case, our patient could not handle more than 16 weeks, even when the dose was lowered. He felt limitative asthenia, and he had thrombocytopenia and neutropenia with subclinical hypothyroidism. This led to multiple appointments and blood tests to monitor his condition, which makes follow-up complicated. With only 16 weeks of treatment, however, he achieved loss of HBsAg, the ultimate treatment endpoint, rarely observed. In fact, HBeAg seroconversion was reported in 22–27% of the patients at the end of 42 weeks' treatment and 29–32% 6 months after, with loss of HBsAg occurring in only 3–5% of patients at end of therapy [11, 12]. Higher rates of seroconversion (67% HBeAg and 8% HBsAg) have been described in a population of 13 children infected with genotype C who received a higher dose of PEG-IFN*α* (180 *µ*g) [13]. We could not, in this case, evaluate HBs seroconversion because our patient had from the start and maintained positive HBsAb. Coexistence between HBsAg and HBsAb has been reported, and it seems to be a rare event. These antibodies are probably unable to neutralize virions or because they target other regions or serotypes or due to immune escape variants not effectively recognized, either way they do not seem to affect clinical course, and these patients should be regarded the same as other HBsAg positive [14].

## 4. Conclusion

We report a singular CHB case with a surprising response to PEG-IFN*α*; with only 16 weeks of treatment, our patient attained loss of HBsAg and sustained off-treatment virological response. Although often disregarded considering its limitative adverse side effects, PEG-INF*α* may achieve similar treatment endpoints to NAs with a finite course of treatment, and it might be an option to patients willing to try it.

The table represents the evolution of HBV markers and hepatic function since the initiation of treatment until 48 weeks (1 year) after its suspension, when sustained off-treatment response was confirmed. As given, there was an overall good response with HBe seroconversion, viral suppression, normalization of transaminases, and ultimately progressive reduction and loss of HBsAg.

## Figures and Tables

**Table 1:** Evolution of patient's HBV markers and hepatic function.

	D0	W12	W14	W16	W24 AT^*∗*^	W48 AT^*∗*^
Viral load (IU/mL)	404 × 10^6^	232	<10	<10	<10	<10
HBsAg HBV (index) (negative if index <1)	598	33.2	−	0.36	0.33	0.24
ALT/AST (U/L)	62/116	227/404	186/309	59/91	38/32	29/29
HBeAg	+	−	−	−	−	−
HBeAb	−	+	+	+	+	+

^*∗*^AT, after treatment.
